# Progress of Ginsenoside Rb1 in neurological disorders

**DOI:** 10.3389/fphar.2024.1280792

**Published:** 2024-01-24

**Authors:** Gongxia Ling, Min Zhang, Chizhang Chen, Yan Wang, Qiqi Gao, Jianshun Li, Hao Yuan, Wenwen Jin, Wei Lin, Lingrong Yang

**Affiliations:** ^1^ Department of Pediatrics, The Second School of Medicine, Wenzhou Medical University, The Second Affiliated Hospital and Yuying Children’s Hospital of Wenzhou Medical University, Wenzhou, Zhejiang, China; ^2^ Department of Clinical Medicine, Pingyang County Traditional Chinese Medicine Hospital, Meizhou, Zhejiang, China; ^3^ Department of Pediatrics, Sichuan Provincial Maternity and Child Health Care Hospital, Chengdu Medical College, Chengdu, Sichuan, China

**Keywords:** Ginsenoside Rb1, pharmacokinetics, actions, mechanisms, neurological disorders

## Abstract

Ginseng is frequently used in traditional Chinese medicine to treat neurological disorders. The primary active component of ginseng is ginsenoside, which has been classified into more than 110 types based on their chemical structures. Ginsenoside Rb1 (GsRb1)—a protopanaxadiol saponin and a typical ginseng component—exhibits anti-inflammatory, anti-oxidant, anti-apoptotic, and anti-autophagy properties in the nervous system. Neurological disorders remain a leading cause of death and disability globally. GsRb1 effectively treats neurological disorders. To contribute novel insights to the understanding and treatment of neurological disorders, we present a comprehensive review of the pharmacokinetics, actions, mechanisms, and research development of GsRb1 in neurological disorders.

## Graphical Abstract

**Figure:**
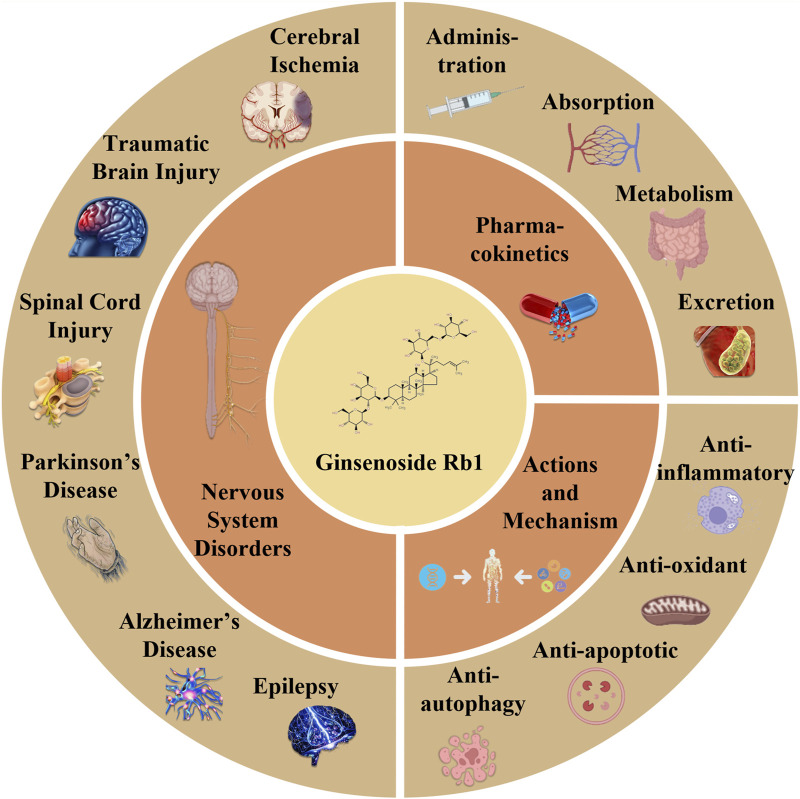
The article reviews the pharmacokinetics, effects, processes, and investigations in developing GsRb1 research. The four components of GsRb1’s pharmacokinetics are administration route, absorption, metabolism, and excretion. GsRb1 can have anti-inflammatory, anti-oxidant, anti-apoptotic, and anti-autophagy properties in the neurological system. Therapeutic applications of GsRb1 include the treatment of epilepsy, Parkinson’s disease, traumatic brain damage, spinal cord injury, cerebral ischemia, and other neurological disorders.

## 1 Introduction

**FIGURE 1:**
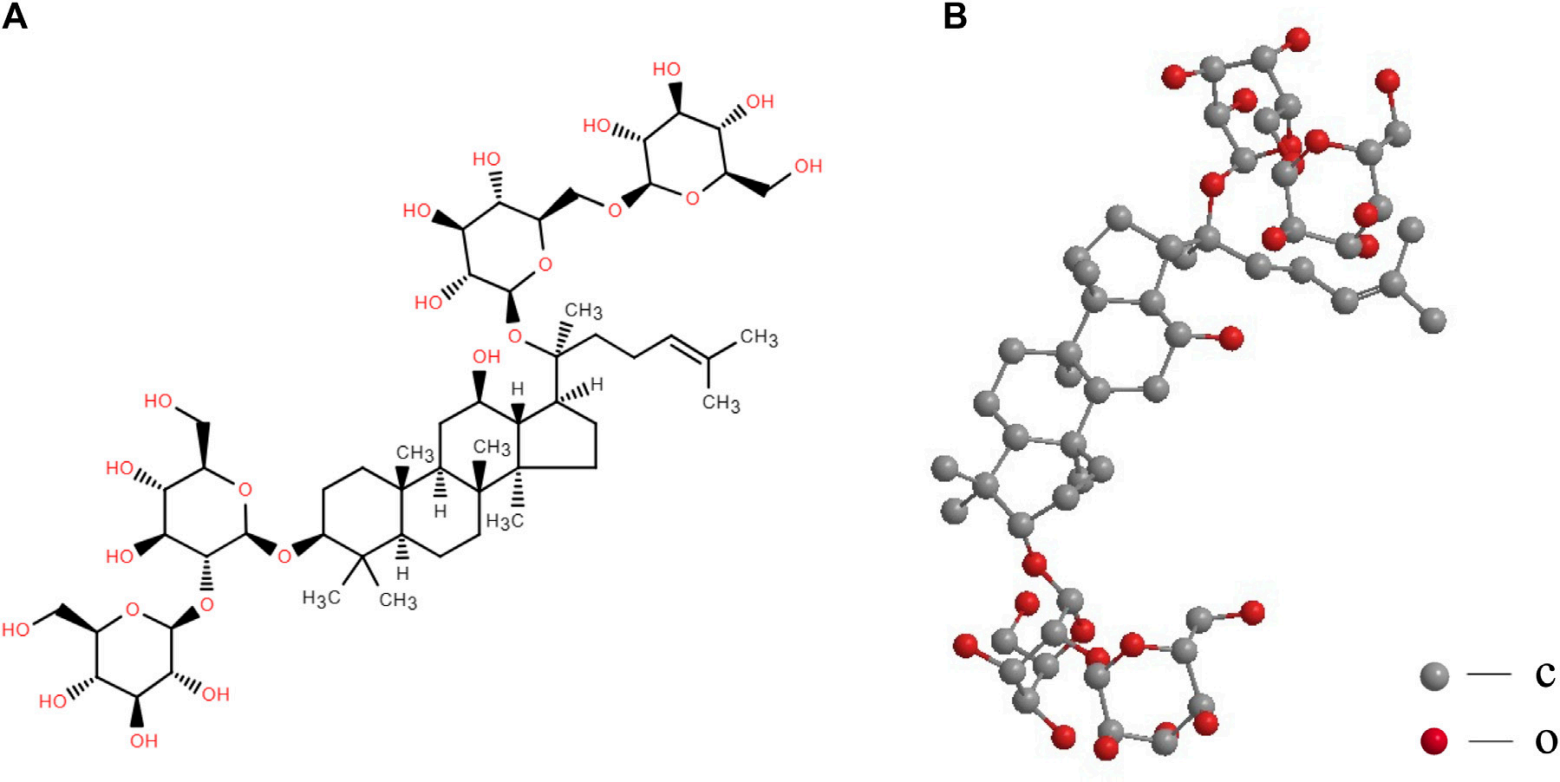
Structural formula **(A)** and molecular stereo structure **(B)** of ginsenoside Rb1. The structural formula of GsRb1 (C_54_H_92_O_23_) is illustrated in **(A)**, with a dammarane (a tetracyclic triterpenoid) at its center and several R groups connecting its upper and lower ends. In **(A)**, the dashed triangles are typically used to represent wedged bonds, indicating that the chemical bond extends outwards from the plane of the paper towards the observer. Solid triangles represent hashed bonds, signifying that the chemical bond extends inward, away from the observer into the plane of the paper. The stereoscopic structure of this molecule, which conceals the hydrogen atoms, is displayed in **(B)**. The oxygen and carbon atoms are represented by the red and gray balls, respectively.

The “king of all herbs” is ginseng, a tonic and medicinal herb (Tao et al., 2023). Traditional Chinese medicine attributes ginseng’s efficacy to prolonging life and replenishing vital energy (Im, 2020). Ginseng’s therapeutic benefits on neurological disorders have been backed by extensive preclinical and clinical data (Mancuso and Santangelo, 2017). The active components of ginseng include saponin, polysaccharide, essential oil, and polypeptide (Ha et al., 2007; Ni et al., 2022; Tao et al., 2023). Ginsenosides consist of 20(S)-protopanaxadiol and 20(S)-protopanaxatriol saponins of the dammarane type (Zhou et al., 2019a). Ginsenoside Rb1 (GsRb1) is a highly prevalent ginsenoside and serves as the primary protopanaxadiol saponin (Figure 1) (Kim et al., 2022; Ni et al., 2022).

GsRb1 can be used therapeutically to treat multi-system illnesses affecting the nervous, cardiovascular, and endocrine systems (Zheng et al., 2017; Zhou et al., 2019b; Gong et al., 2022). GsRb1 has been found to exhibit several biological activities, particularly in the nervous system. These activities can penetrate the blood-brain barrier and exert neuroprotective effects such as anti-inflammatory, anti-oxidant, anti-apoptotic, and anti-autophagy (Kim, 2012; Kim et al., 2013; Ong et al., 2015; Zhou et al., 2019b). Recent studies have suggested that GsRb1 can inhibit inflammation, oxidative stress, and excitotoxicity, attenuate neuronal damage, and promote neuronal cell repair to treat neurological diseases (Kiefer and Pantuso, 2003; Yang JE. et al., 2020; Shi et al., 2020). These findings suggest that GsRb1 may be more effective in treating epilepsy, Alzheimer’s disease (AD), and Parkinson’s disease (PD).

Cerebrovascular disease, spinal cord lesions, epilepsy, and neurodegenerative disorders are among the most prevalent neurological conditions in humans (Gooch et al., 2017). Lifelong medication is mandatory for most patients, resulting in substantial adverse effects (Cai et al., 2020). Studies have demonstrated that GsRb1 is a prospective new star in the pharmacological treatment of neurological disorders because it has good pharmacokinetics, numerous neuroprotective properties, and fewer side effects (Gong et al., 2022). The present review provides an overview of the pharmacokinetics, results, mechanisms, and progress of research on GsRb1 in neurological disorders to generate novel ideas for the treatment and study of such conditions.

## 2 Pharmacokinetics of ginsenoside Rb1

**FIGURE 2:**
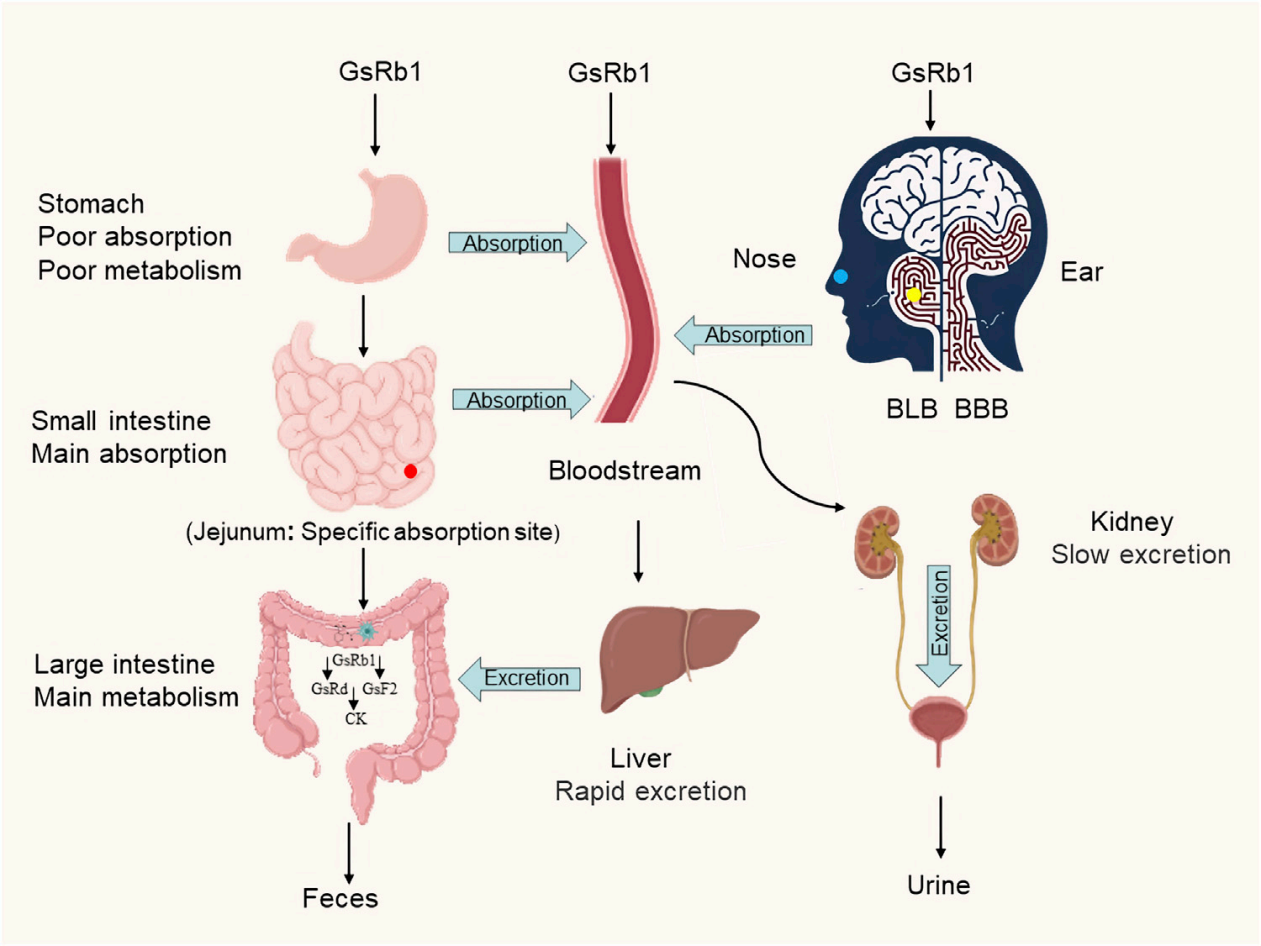
Metabolic pathways of ginsenoside Rb1. GsRb1 can enter the body and circulate in the bloodstream through various administration routes, including oral ingestion, intravenous injection, intratympanic administration (indicated by yellow dots representing the location of the middle ear), and intranasal administration (represented by blue dots indicating the nasal administration site). After oral ingestion, GsRb1 is characterized by complete intestinal absorption, with the jejunum showing a specific absorption site (indicated by red dots representing the location of the jejunum). Intratympanic administration allows GsRb1 to bypass the BLB and BBB, resulting in higher drug concentrations in the inner ear and the brain. Once GsRb1 enters the intestines, it undergoes rapid metabolism and breakdown facilitated by gut microbiota, leading to the formation of GsRd, GsF2, and CK. The primary metabolic site is the colon (major metabolic site), followed by the stomach (minor metabolic site). GsRb1 is primarily excreted through rapid biliary excretion and slow renal excretion. Abbreviation: GsRb1, Ginsenoside Rb1; BLB, Blood-Labyrinth Barrier; BBB, the Blood-Brain Barrier; GsRd, Ginsenoside Rd; GsF2, Ginsenoside F2; CK, Compound K.

**FIGURE 3:**
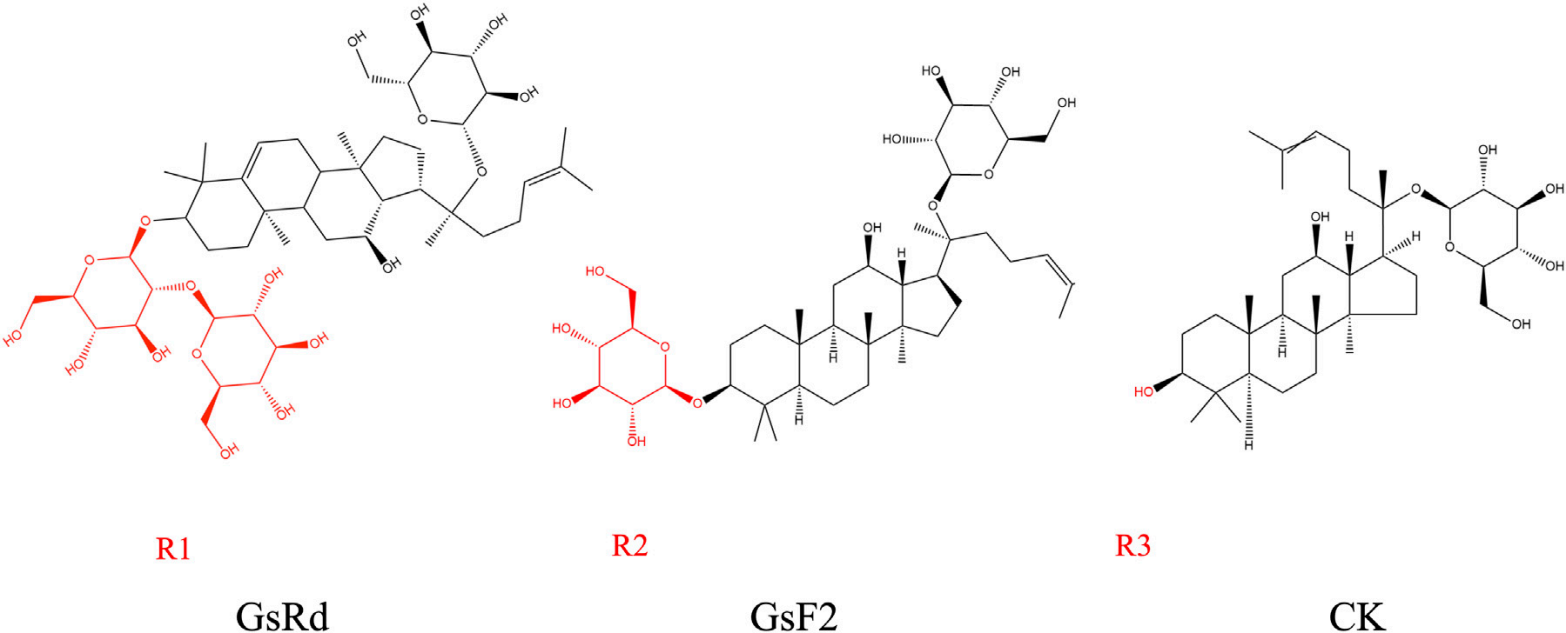
Metabolites of ginsenoside Rb1. GsRb1 undergoes primary metabolic conversion into GsRd, GsF2, and CK. These three compounds share structural similarities, with the main distinction lying in their respective R groups, as highlighted in red in each molecular structure diagram. GsRd has an R group denoted as R1, which is a disaccharide structure formed by a glycosidic bond between two monosaccharides. GsF2 possesses an R group known as R2, which is a monosaccharide structure. CK features an R group designated as R3, which consists of a hydroxyl group. Abbreviation: GsRb1, Ginsenoside Rb1; GsRd, Ginsenoside Rd; GsF2, Ginsenoside F2; CK, Compound K.

GsRb1 has better pharmacokinetics and can be delivered into the human body through various methods. Its absorption, metabolism, and excretion follow a certain pattern (Figure 2). Following gavage, GsRb1 is marginally absorbed in the stomach (Kim, 2018). Most GsRb1 is then absorbed in the entire intestine, with the jejunum having specific absorption sites and a higher concentration of the drug than in the ileum and duodenum, and the intestines are then passively absorbed into the blood (Li et al., 2009; Kim, 2018). A study demonstrated that the absorption, rate, cumulative percent absorption, and half-life did not significantly correlate with the potential of hydrogen (pH) of the intestinal segment’s circulating fluid (Li et al., 2009). Human intestinal flora can quickly metabolize and degrade GsRb1 after it enters the gastrointestinal system, and the large intestine is the primary metabolic location, followed by the stomach (Bae et al., 2000; Kim, 2018; Zhao et al., 2023). *In vivo*, GsRb1 was first transformed into ginsenosides Rd (GsRd) and ginsenosides F2 (GsF2) and then into ginsenoside compound K (CK) (Figure 3), also known as 20-O-glucopyranosyl-20 (S) protopanaxadiol (Kim, 2013; Chen et al., 2015). GsRb1 and compound K have neuroprotective properties. After administering 9 g of GsRb1 orally to ten healthy Korean male subjects, the researchers quickly identified GsRb1 in the plasma of the subjects, which reached its Maximum Concentration (C_max_) of (3.94 ± 1.97) ng/mL in approximately 5 h and its half-life (t_1/2_) of (58.47 ± 14.28) h (Kim, 2013). After oral administration of GsRb1 to fasted Sprague-Dawley rats (weighing 300–320 g, obtained from the Laboratory Animal Center of Fudan University, Shanghai, China) at a dose of 600 mg/kg using a 120 mg/mL panax notoginseng saponins (PNS) aqueous solution, the serum concentration of Rb1 peaked at 1.5 h, reaching 47.13 mg/mL, and remained elevated for 72 h, resulting in an absolute oral bioavailability of 4.35% (Xu et al., 2003; Won et al., 2019). Furthermore, GsRb1 may boost its bioavailability when paired with the P-glycoprotein (P-gp) inhibitor verapamil because it serves as a substrate for P-gp, a significant transporter protein linked to drug transport in cell membranes (Li et al., 2009). GsRb1 has a low bioavailability as it is excreted in the stomach, colon, and liver; however, increasing the permeability of its membranes might improve its absorption (Han et al., 2006). Ginsenosides are excreted through the kidneys and biliary tract, and GsRb1 passes via both sluggish renal excretion and quick biliary excretion (Won et al., 2019). When Sprague-Dawley rats weighing 250–300 g, sourced from the Laboratory Animal Center of Fudan University in Shanghai, China, were intravenously administered a 50 mg/mL PNS physiological saline solution at a dose of 50 mg/kg, bile excretion reached equilibrium and entered a plateau phase at 6 h (Han et al., 2007). At 10 h after administration, GsRb1 exhibited a bile excretion rate of (3.94 ± 1.49)% of the intravenous dose, with an absolute bioavailability of 65.77% following portal vein injection, a mean residence time (MRT) of 24.6 h, and a high (>80%) plasma protein binding rate. In contrast to oral administration, intravenous (IV) administration showed lower bile excretion but higher absolute bioavailability and plasma protein binding rates, indicating that IV administration is the preferred route for GsRb1 for therapeutic purposes (Han et al., 2006; Han et al., 2007). Intratympanic (IT) is a novel mode of administration compared with IV, as it can disseminate GsRb1 to the inner ear and then transport it to the brain (Chen et al., 2007; Long et al., 2014). This delivery approach circumvents both the Blood-Labyrinth Barrier (BLB) and the Blood-Brain Barrier (BBB), allowing the drug to reach its target more directly at lower doses. As a result, it achieves higher drug concentrations in the inner ear and the brain while minimizing systemic side effects (Long et al., 2014). According to the data, C_max_ was 1.5 and 0.4 times higher in cerebrospinal fluid and brain tissue, respectively, after IT than after IV, and the area under the curve (AUC) was 0.5 and 1.2 times higher, respectively, after IT than after IV, but C_max_ and AUC in plasma were 45.9% and 33.1% lower, respectively, after IT than after IV (Chen et al., 2011). Additionally, intranasal administration is another efficient way to reach the central nervous system and is superior to other approaches because of its ease of usage, quick absorption, and avoidance of the initial excretion. Intranasal delivery of GsRb1 rapidly penetrates the brain within 5 min, and local bioavailability in specific brain areas is likewise significantly higher than that in plasma (Lu et al., 2011).

Furthermore, GsRb1 can be converted into the stable chemical derivative Rb1, dihydroginsenoside Rb1 (dgRb1), and pertinent studies have demonstrated that the effective dose of dgRb1 is ten times lower than that of GsRb1 (Sakanaka et al., 2007). Additionally, IV infusion of an effective dose of dgRb1 does not affect systemic parameters like brain temperature, blood pressure, or cerebral blood flow when repairing damaged neurons (Sakanaka et al., 2007). Panax ginseng Meyer Herbal Preparation HRG80 has garnered widespread recognition both domestically and internationally, with demonstrated significant efficacy, particularly in preventing and alleviating stress-induced cognitive function impairment in healthy individuals (Mariage et al., 2020). Certain GsRb1 class drugs, such as Vitacost Ginseng Extract Complex, have received drug approval numbers and have been proven to enhance energy levels, attention, and memory. These medications boast unique formulations and ingredients, with GsRb1 being acknowledged for its outstanding stress resistance and neuroprotective properties, contributing to memory enhancement, improved attention, and the promotion of cognitive abilities (Ahmed et al., 2016; Jakaria et al., 2018). Nowadays, oral medications predominate; therefore, developing strategies that enhance a drug’s oral bioavailability is crucial. However, GsRb1 has a low oral bioavailability, which significantly reduces its efficacy and hinders the continued exertion of clinical efficacy. GsRb1 possesses pharmacological effects that extend beyond its single-molecule form; it can be converted to dgRb1 to play an important role; accordingly, it has application prospects in preventing and treating neurological disorders. IV, IT, and intranasal administrations currently provide more options for entering GsRb1 into the brain.

## 3 Effect and mechanism of ginsenoside Rb1

**FIGURE 4:**
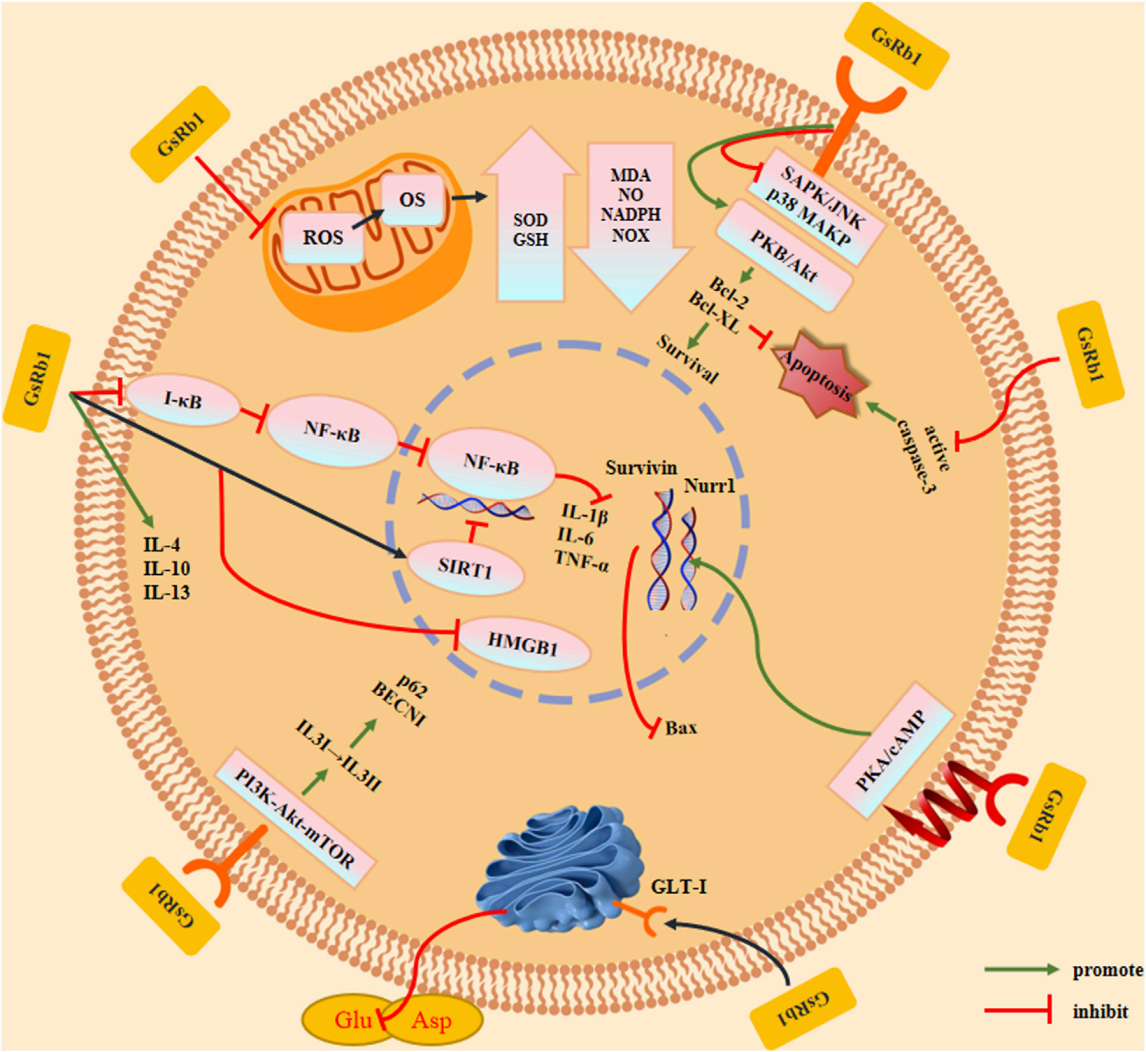
Effects and mechanisms of ginsenoside Rb1. GsRb1 can act as an anti-inflammatory, anti-oxidant, anti-apoptotic, and anti-autophagic factor in cells. By controlling IkB-α to prevent the formation of NF-κB dimers and activation of SIRT1, GsRb1 reduces inflammation by inducing the production of anti-inflammatory factors (IL-4, IL-10, and IL-13), thereby inhibiting HMGB1 inflammatory signaling and blocking the synthesis of inflammatory factors (IL-1β, IL-6, and TNF-α). GsRb1 modulates ROS levels in mitochondria that affect OS by upregulating SOD and GSH expression, downregulating MDA, NO, NADPH, and NOX expression, and lowering the neurotoxicity brought on by free radicals and other substances. GsRb1 enhanced cell survival by upregulating the expression of Bcl-2 and Bcl-XL, decreasing caspase-3 activation to limit apoptosis, and decreasing phosphoprotein expression of SAPK/JNK or p38 MAPK. These changes also increased the expression of Bcl-2 and Bcl-XL. Concurrently, GsRb1 controls the PKA/cAMP signaling pathway to reduce Nurr1 levels and increase Survivin expression, which prevents apoptosis. GsRb1 also increases GLT-1 expression on vesicle membranes and decreases the amount of Glu and Asp in tissues to prevent amino acid excitotoxicity. GsRb1 reduces the autophagy marker protein BECN1, downregulates the autophagic breakdown substrate p62, and significantly attenuates hyperactivated autophagy in cells. It also activates PI3K-Akt-mTOR *in vivo* and changes LC3 from type I to type II. Abbreviation: Akt, protein kinase B; Bcl-2, B-cell lymphoma 2; Bcl-XL, B-cell lymphoma-extra large; BECN1, Beclin 1; cAMP, Cyclic adenosine monophosphate; GLT-1, Glutamate transporter 1; Glu, Glutamate; GSH, Glutathione; IL, Interleukin; HMGB1, High mobility group box protein 1; JNK, c-Jun N-terminal kinase; LC3, Light chain 3; MDA, Malondialdehyde; mTOR, Mammalian target of rapamycin; NADPH, Nicotinamide adenine dinucleotide phosphate; NF-κB, Nuclear factor kappa B; NO, Nitric oxide; NOX, NADPH oxidase; Nurr1, Nuclear receptor-associated protein 1; OS, Oxidative stress; p38 MAPK, p38 mitogen-activated protein kinases; PI3K, Phosphoinositide 3-kinase; PKA, Protein kinase A; ROS, Reactive oxygen species; SAPK, Stress-activated protein kinase; SIRT1, Sirtuin 1; SOD, Superoxide dismutase; TNF-α, Tumor necrosis factor alpha.

GsRb1 has been shown to possess a diverse array of biological activities, including the capacity to cross the blood-brain barrier and produce neuroprotective effects, such as anti-inflammatory, anti-oxidant, anti-apoptotic, and anti-autophagic effects (Figure 4; Table 1) (Lin et al., 2022; Zhang M. et al., 2023; Hu et al., 2023; Jiang et al., 2023).

**TABLE 1:** Effects and mechanisms of ginsenoside Rb1.

Effect	Mechanism	References
Anti-inflammation	1. Regulating I-κB to block NF-κB dimerization and activate SIRT1	Li et al. (2007), Wang et al. (2011), Zhang et al. (2018a), Liu et al. (2018), Jiang et al. (2023)
	2. Inhibiting the synthesis of inflammatory cytokines (IL-1β, IL-6, TNF-α)	
	3. Inducing the synthesis of anti-inflammatory cytokines (IL-4, IL-10, IL-13)	
	4. Suppressing HMGB1 inflammatory signal transduction	
Anti-oxidant	1. Regulating ROS levels in mitochondria	Dong et al. (2017), Shi et al. (2018), Zhou et al. (2019b), Xie et al. (2021)
	2. Increasing the expression of SOD and GSH.	
	3. Reducing the expression of MDA, NO, NADPH, and NOX.	
	4. Mitigating neurotoxicity caused by free radicals	
Anti-apoptotic	1. Decreasing the expression of phosphorylated proteins in the SAPK/JNK or p38 MAPK pathways	Hashimoto et al. (2012), Ahmed et al. (2016), Chen et al. (2016), Wang et al. (2017), Ye et al. (2019), Yang et al. (2020b)
	2. Increasing the expression of phosphorylated proteins in the PKA/cAMP and ERK1/2 pathways	
	3. Upregulating the expression of Bcl-2 and Bcl-XL.	
	4. Reducing caspase-3 activation to inhibit cell apoptosis	
	5. Modulating Nurr1 levels by regulating the PKA/cAMP signaling pathway	
	6. Promoting the expression of Survivin	
	7. Enhancing the expression of GLT-1 on vesicle membranes, reducing the levels of Glu and Asp in tissues	
Anti-autophagy	1. Activating the PI3K-Akt-mTOR pathway	Luo et al. (2014), Zhou et al. (2018), Liu et al. (2020), Zou et al. (2022)
	2. Converting LC3 from type I to type II.	
	3. Decreasing the autophagy marker protein BECN1	
	4. Downregulating the autophagic substrate p62	

Abbreviation: Akt, protein kinase B; asp, Aspartate; Bcl-2, B-cell lymphoma 2; Bcl-XL, B-cell lymphoma-extra large; BECN1, Beclin 1; cAMP, cyclic adenosine monophosphate; ERK, extracellular regulated protein kinase; GSH, glutathione; I-κB, Nuclear factor kappa-B, kinase subunit Alpha; IL, interleukin; HMGB1, High mobility group box protein 1; JNK, c-Jun N-terminal kinase; LC3, Light chain 3; MDA, malondialdehyde; mTOR, mammalian target of rapamycin; NADPH, nicotinamide adenine dinucleotide phosphate; NF-κB, Nuclear factor kappa B; NO, nitric oxide; NOX, NADPH, oxidase; Nurr1, Nuclear receptor-associated protein 1; p38 MAPK, p38 mitogen-activated protein kinases; PI3K, Phosphoinositide 3-kinase; PKA, Protein kinase A; ROS, reactive oxygen species; SAPK, Stress-activated protein kinase; SIRT1, Sirtuin 1; SOD, superoxide dismutase; TNF-α, tumor necrosis factor alpha.

### 3.1 Anti-inflammation

The anti-inflammatory actions of GsRb1 are primarily manifested in the suppression of inflammatory factor production, activation of anti-inflammatory factor synthesis, and inhibition of inflammatory conduction pathways in the inflammatory response. First, GsRb1 prevents the production of inflammatory factors by controlling two key inflammatory regulators, cyclooxygenase-2 (COX-2) and nuclear factor-kappa B (NF-κB) (Wang et al., 2011). According to studies, GsRb1 controls NF-κB in two ways: on the one hand, by controlling Inhibitor of nuclear factor kappa-B Kinase Subunit Alpha (IkB-α), which prevents the synthesis of NF-κB dimers, and on the other hand, by reducing inflammation (Li et al., 2007; Wang et al., 2011). By contrast, GsRb1 downregulates the expression of interleukin (IL)-1β (IL-1β), IL-6, and tumor necrosis factor-α (TNF-α) in ischemic brain injury and decreases the production of inflammatory factors by activating silent information regulator 1 (SIRT1) (Zhou et al., 2018; Cheng et al., 2019; Qi et al., 2020; Zheng et al., 2020). Furthermore, GsRb1 can increase anti-inflammatory M2 macrophage polarization by promoting the expression of the anti-inflammatory cytokines, such as IL-4, IL-10, and IL-13, as well as two conventional M2 macrophage markers, arginase-I (Arg-I), and macrophage mannose receptor (Chen et al., 2015; Zhang X. et al., 2018). Inhibiting inflammatory high mobility group box protein 1 (HMGB1) signaling, a frequently occurring molecule that exacerbates inflammatory damage, may allow GsRb1 to have anti-inflammatory effects (Liu et al., 2018). HMGB1 is a highly conserved non-histone deoxyribonucleic acid (DNA)-binding nucleoprotein (Chen et al., 2004). Inflammation is a common underlying mechanism in various conditions such as brain injury, cerebral ischemia, aging, and neurodegenerative diseases. GsRb1 demonstrates its anti-inflammatory effects through multiple pathways, making it a valuable asset in the treatment of neurological disorders. GsRb1’s actions are governed by a complex molecular regulatory network, and future research can delve deeper into understanding the intricate interactions among these mechanisms, providing a more comprehensive grasp of its anti-inflammatory properties. Moreover, GsRb1 not only suppresses the synthesis of pro-inflammatory cytokines but also facilitates the expression of anti-inflammatory cytokines like IL-4, IL-10, and IL-13. This pivotal role in maintaining the balance within the inflammatory response warrants further investigation. Subsequent studies can explore how GsRb1 precisely modulates the equilibrium between pro-inflammatory and anti-inflammatory factors, thus paving the way for more targeted anti-inflammatory treatments. In summary, GsRb1, with its multifaceted mechanisms and effects as an anti-inflammatory agent, offers promising avenues for future research in diseases associated with inflammation. Researchers can delve deeper into these mechanisms to develop more effective treatment strategies, fully unlocking the medical potential of GsRb1.

### 3.2 Anti-oxidant

GsRb1 has an anti-oxidant effect on oxidative stress (OS) by controlling the amounts of reactive oxygen species (ROS) in mitochondria, reducing the neurotoxicity induced by free radicals (Ni et al., 2022). ROS, including oxygen-containing radicals, oxygen-free radicals, non-radical derivatives, and hydroperoxides, are produced mainly by oxidative stress in mitochondria (Zhou et al., 2019b). Neurological disorders can develop because of severe OS that can cause brain cell malfunctions, such as proliferative arrest, aging, apoptosis, and necrosis (Rottenberg and Hoek, 2017). GsRb1 can directly reduce ROS levels in mitochondria or promote the production and transformation of mitochondrial energy through related metabolic enzymes, specifically by enhancing anti-oxidant enzyme activity and inhibiting oxidase activity to inhibit ROS accumulation in mitochondria during oxidative stress, such as increasing anti-oxidant enzyme superoxide dismutase (SOD) levels and increasing the expression of ROS scavenger glutathione (GSH), exerting anti-oxidant effects and reducing malondialdehyde (MDA), nitric oxide (NO) and nicotinamide adenine dinucleotide phosphate (NADPH) expression, markers of oxidative stress (Dong et al., 2017; Shi et al., 2018; Zhou et al., 2019b; Liu et al., 2023). GsRb1 can also boost mitochondrial membrane potential, protect the normal oxidative stress function of mitochondria, and maintain the integrity of mitochondrial function, all of which reduce mitochondrial damage in various neurons (Cheng et al., 2019). GsRb1 also inhibits the primary ROS source *in vivo* and lowers ROS generation by negatively regulating nicotinamide adenine dinucleotide phosphate oxidase (NOX)-1, NOX-2, and NOX-4 activity following ischemia (Dong et al., 2017; Xie et al., 2021).

An increasing body of research indicates that as individuals age, the functionality of the body’s antioxidant systems gradually diminishes. Meanwhile, oxidative stress, driven by ROS, plays a pivotal role in the pathogenesis of various neurological disorders. The protective effects of GsRb1 on mitochondria may constitute a critical aspect of its antioxidant capabilities. Furthermore, GsRb1 is likely to mitigate oxidative stress through multiple pathways, negatively regulating the activity of NADPH oxidases, thereby reducing ROS production. This may represent another key mechanism underlying its antioxidant properties. In the future, a more in-depth exploration into how GsRb1 influences the generation and accumulation of ROS within mitochondria can be pursued. Additionally, investigating the interactions between GsRb1 and various subtypes of NADPH oxidases, as well as its involvement in mitochondrial antioxidant processes, holds promising prospects. Summarizing these insights and perspectives will provide valuable directions for further comprehensive research into GsRb1’s antioxidant effects and its potential applications in the treatment of neurological disorders. Such studies will contribute to a deeper understanding of GsRb1’s mechanisms in neuroprotection.

### 3.3 Anti-apoptotic

GsRb1 prevents neuronal death by controlling gene and enzyme expression, encouraging neurotrophin release, and blocking the excitotoxic effects of amino acids and calcium overload. In apoptosis caused by 1-methyl-4-phenylpyridine (MPP+), GsRb1 effectively reduced MPP + -induced caspase-3 activation and DNA fragmentation and increased B-cell lymphoma-extra large (Bcl-XL) gene expression in pheochromocytoma cells (PC12) (Undifferentiated rat pheochromocytoma PC12 cells were cultured in 75 cm^2^ tissue culture flasks. Cells were maintained in Dulbecco’s modified Eagle’s medium containing 5% heat-inactivated Fetal Bovine Serum and 10% heat-inactivated Horse Serum supplemented with 100 U/mL penicillin and 100 μg/mL streptomycin in a water-saturated atmosphere of 5% Carbon Dioxide in air at 37°C.), which significantly improved cell viability (Zhang et al., 2006; Hashimoto et al., 2012; Zhang et al., 2012; Dong et al., 2022). Furthermore, GsRb1 can suppress microglial apoptosis, increase neuronal recovery *in vitro*, and upregulate the expression of anti-apoptotic proteins, including B-cell lymphoma 2 (Bcl-2) (Lee et al., 2018). By lowering the phosphorylated protein expression of stress-activated protein kinase/c-Jun N-terminal kinase (SAPK/JNK) or p38 mitogen-activated protein kinases (p38 MAPK), GsRb1 can increase the phosphorylated protein expression of protein kinase B (Akt/PKB) and extracellular regulated protein kinase (ERK) 1/2 (ERK1/2) (Hashimoto et al., 2012; Chen et al., 2016). Moreover, GsRb1 reduces the expression of pro-apoptotic Bax protein by regulating the levels of nuclear receptor-associated protein 1 (Nurr1), an essential factor in inhibiting microglial apoptosis, and by regulating the protein kinase A/cyclic adenosine monophosphate (PKA/cAMP) signaling pathway (Lee et al., 2018). GsRb1 can also prevent apoptosis by encouraging the expression of the apoptosis suppressor gene Survivin (Ye et al., 2019). GsRb1 reduces the levels of glutamate (Glu) and aspartate (Asp) in brain tissue by increasing the expression of glutamate transporter 1 (GLT-1) on the vesicle membrane (Wang et al., 2017; Yang F. et al., 2020). Furthermore, GsRb1 prevented neurons from Glu toxicity, inhibited L-type voltage-gated calcium channels in hippocampal neurons in a dose-dependent manner, decreased cell death, and increased the longevity of surviving neurons, exerting a protective effect on cortical neurons and dopaminergic neurons (Lin et al., 2012; Ahmed et al., 2016). In the artificial abnormal microenvironment caused by microperfusion of L-glutamate and calcium ions (Ca^2+^), the protein kinase B/mammalian target of rapamycin/phosphatase and tensin homolog signaling pathway (Akt/mTOR/PTEN) signaling pathway is involved in cell growth and proliferation and inhibits apoptosis. GsRb1 increases the expression of phosphorylated-Akt (p-Akt) and phosphorylated-mTOR (p-mTOR) and decreases phosphorylated-PTEN (p-PTEN) *in vitro* and *in vivo*, thereby alleviating neuronal injury after abnormal microperfusion, proving that GsRb1 inhibits amino acid excitotoxicity and calcium overload (Guo et al., 2018). In the context of preventing neurological disorders, the control of cell apoptosis can be instrumental in reducing the incidence of these diseases and, potentially, in slowing down or preventing their progression. It is imperative to conduct further in-depth research into the influence of GsRb1 on apoptotic signaling pathways, encompassing its regulatory mechanisms involving key molecules such as Akt/mTOR, PKA/cAMP, and the Bcl-2 family. Through comprehensive molecular-level investigations, we can unravel the variations in the effects of GsRb1 across diverse cell types and disease models. This, in turn, will provide a robust scientific basis for the development of more precise therapeutic strategies. GsRb1 manifests its anti-apoptotic effects through multiple pathways, effectively inhibiting neuronal apoptosis and alleviating damage to the nervous system. These actions contribute to the maintenance of normal bodily functions, underscoring its substantial potential value in the prevention and treatment of neurological disorders.

### 3.4 Anti-autophagy

GsRb1 has anti-autophagy effects and can activate the phosphoinositide 3-pinase/protein pinase B signaling pathway (PI3K/Akt) pathway *in vivo* (Zhou et al., 2019b; Qin et al., 2021). Excessive cellular activation of autophagy can be significantly decreased in the presence of a PI3K activator, Akt activator, or mTOR activator (Guan et al., 2017). The mechanism underlying this decrease in autophagy is that GsRb1 converts microtubule-associated protein light chain 3 (LC3) from type I to type II, significantly increases the expression ratio of ILC3-II/LC3-I (Zhou et al., 2018; Zou et al., 2022), decreases the autophagy marker protein beclin1 (BECN1) (Chen et al., 2010; Luo et al., 2014), and downregulates the autophagic degradation substrate sequestosome 1 (p62) (Li et al., 2017; Liu et al., 2020), thereby inhibiting excessive autophagy in neurons (He et al., 2017). Further investigation is necessary to uncover the precise mechanisms by which GsRb1 activates the PI3K/Akt pathway, its interactions with key molecules such as PI3K, Akt, mTOR, as well as its mechanisms for diminishing the autophagic marker Beclin1 and reducing the autophagic substrate p62. In the nervous system, although autophagy is generally protective, it accelerates the injurious degeneration of neurons after acute injury to the nervous system (Choi et al., 2022), whereas GsRb1 can resist autophagy, bringing potential novel multi-targeted therapeutic strategies for these diseases.

## 4 Ginsenoside Rb1 and neurological system disorders

Previous research has demonstrated that GsRb1 has various effects, such as anti-inflammatory, anti-oxidant, anti-apoptotic, and anti-autophagy in the nervous system, and can play an essential role in developing and progressing neurological disorders. Some studies have also demonstrated that GsRb1 is a potential therapeutic agent for various neurological disorders, such as cerebral ischemia, traumatic brain injury, spinal cord injury, PD, AD, and epilepsy (Table 2).

**TABLE 2:** Effects and mechanisms of GsRb1 in neurological disorders.

Disease	Action	Mechanism	Effect	References
Cerebral Ischemia	Anti-inflammatory, anti-autophagic, antiapoptotic, anti-oxidant	Reduce inflammatory factors, increase anti-inflammatory factors, inhibit mitochondrial apoptosis, inhibit excessive autophagy in nerves, promote the release of BDNF and GDNF, and inhibit ROS production	Protect blood-brain barrier and nerve cell function, reduce ischemia-reperfusion injury, and improve the motor score and cognitive impairment	Gao et al. (2010), Cheng et al. (2019), Zeng et al. (2022)
Traumatic Brain Injury	Antiapoptotic, anti-autophagic	Upregulate ERK1/2 phosphorylation and decrease Cx40 expression	Reduce brain edema, reduce basilar artery vasospasm and lumen thickness, and improve neurobehavior after brain injury in rats	Li et al. (2011), Zhang et al. (2012), Guo et al. (2014)
Spinal Cord Injury	Anti-inflammatory, anti-autophagic, anti-oxidant, antiapoptotic	The substantial decrease in nerve action potential	Inhibit neuronal injury, improve neurological function, and relieve pain	Huang et al. (2015), Wang et al. (2018)
Parkinson’s Disease	Antiapoptotic	Upregulate GABAARα1 expression in cells, increase PSD-95 expression, and upregulate glutamate transporter protein expression	Improve GABAergic transmission, reduce glutamate excitotoxicity, and improve memory impairment and dyskinesia	Zhang et al. (2018b), Qu et al. (2019)
Alzheimer’s Disease	Antiapoptotic, anti-oxidant, anti-inflammatory	Reduce calpain and p25 expression levels, attenuate tau hyperphosphorylation in cortical neurons, and reduce ROS	Increase microtubule stability, maintain intracellular calcium homeostasis, protect nerve cells, and improve learning and memory behaviors	Humbert et al. (2000), Chen et al. (2008)
Epilepsy	Anti-inflammatory, anti-autophagic, anti-oxidant	Upregulate MDA and Bcl-2 expression; decrease GSH, inducible NOS (iNOS), and LC3 expression; increase Nrf2 and heme oxygenase-1 (HO-1) gene expression	Reduce seizure duration, prolong latency to reoccurrence, reduce brain damage from epilepsy, and improve cognitive dysfunction	Shi et al. (2018), Zeng et al. (2018), Chen et al. (2019)

Abbreviation: Bcl-2, B-cell lymphoma 2; BDNF, Brain-derived neurotrophic factor; Cx40, Connexin 40; ERK, Extracellular signal-regulated kinase; GABAARα1, Gamma-Aminobutyric acid receptor Alpha 1; GDNF, Glial cell line-derived neurotrophic factor; GSH, glutathione; HO-1, Heme oxygenase-1; iNOS, inducible nitric oxide synthase; LC3, Light chain 3; MDA, malondialdehyde; Nrf2: Nuclear factor erythroid 2-related factor 2; PSD-95, Postsynaptic density protein 95; ROS, reactive oxygen species.

### 4.1 Ginsenoside Rb1 and cerebral ischemia

Cerebral ischemia is currently associated with the most significant fatality and disability rates among cardiovascular disorders (Yang et al., 2022). It broadly refers to a lack of cerebral blood flow that hinders the support of brain tissue metabolism. Its progression can lead to neurosensory and motor dysfunction, which includes brain damage and aberrant changes in brain function (Zhang et al., 2022). Its causes are numerous and complicated, including smoking, diabetes, hypertension, and atherosclerosis (Sveinsson et al., 2014). Because inflammation can result in subsequent injury following cerebral ischemia, the pathological process of cerebral ischemia is complicated (Zeng et al., 2022). GsRb1 can prevent inflammation, safeguard the tight junctional activity of endothelial cells, and preserve the BBB structural integrity (Chen et al., 2015; Dong et al., 2017). GsRb1 attenuates neurological impairment, lowers the area of cerebral infarction, and enhances anti-inflammatory factors in localized cerebral ischemia (Liu et al., 2013; Zeng et al., 2022). Besides, it lessens inflammation-related redness, swelling, and heat discomfort (Yang F. et al., 2020). Multiple pathways, mostly mitochondria-mediated apoptosis, can be used by cerebral ischemia to cause apoptosis (Zhou et al., 2019b). GsRb1 can protect the structure and function of mitochondria, inhibit neuronal apoptosis and excessive autophagy, and attenuate neurological injury caused by ischemia-reperfusion and autophagic neuronal death induced by ischemic injury (Yang et al., 2008; Luo et al., 2014; Zhao D. et al., 2018). GsRb1 preserved neuronal cells, increased levels of brain-derived neurotrophic factor (BDNF) and glial cell-derived neurotrophic factor (GDNF), and greatly enhanced sensorimotor scores in rats (Adult male Wistar rats weighing 250–300 g were utilized in all experiments. These inbred strain rats were sourced from the Animal House Center at the Third Military Medical University in Chongqing, China. Middle cerebral artery occlusion was induced using the intraluminal vascular occlusion method.) suffering from cerebral ischemic stroke (Yuan et al., 2007; Gao et al., 2010; Jiang et al., 2013). The microenvironment of the central neuron changes when there is cerebral ischemia, which causes neuronal injury (Wang et al., 2017). GsRb1 can influence the phosphorylation of mTOR/PTEN under abnormal microenvironments, reverse pyramidal cell injury in the cornu ammonis (CA) 1 (CA1) region of the hippocampus, attenuate neuronal damage, and lessen cognitive dysfunction following cerebral ischemia (Guo et al., 2018). Regulating oxidative stress is an effective therapeutic method to protect neurons from cerebral ischemia-reperfusion (Wu et al., 2020). GsRb1 can be a protective agent against hypoxia, protecting mitochondrial function, decreasing ROS generation, and enhancing neuronal survival (Park et al., 2005; Xu et al., 2020).

Cheng Z et al. found that GsRb1 is a better ginsenoside to reduce intracellular neuronal apoptosis by comparing the protective effects of six ginsenosides (Rg1, Rb1, Rh2, Rg3, Rg5, and Re) on cerebral ischemia, which can significantly reduce cerebral ischemic injury and neurological deficit and may be used as an effective drug to treat cerebral ischemia (Cheng et al., 2019). Additionally, GsRb1 can be therapeutically valuable up to 24 h post-stroke, and it works well to increase the likelihood of long-term neurological recovery by activating the cyclic adenosine monophosphate/protein kinase A/cAMP response element-binding protein (cAMP/PKA/CREB) signaling pathway *in vivo* and encouraging axonal regeneration in the event of delayed stroke treatment (Yoshikawa et al., 2008; Gao et al., 2020). In conclusion, because GsRb1 can lessen the severity of ischemia injury, attenuate neuronal damage, and enhance cognitive function, it is anticipated to be a viable therapeutic agent in cerebral ischemic illnesses.

### 4.2 Ginsenoside Rb1 and traumatic brain injury

Traumatic brain injury (TBI) typically results from a primary injury caused by physical force to the head area, which subsequently develops into secondary injuries that cause neurological abnormalities that may be transient or permanent (Galgano et al., 2017), such as cerebral edema and cerebral hemorrhage (Hackenberg and Unterberg, 2016). Connexin 40 (Cx40) expression and the severity of TBI are firmly connected, and research has demonstrated that GsRb1 can significantly increase ERK1/2 phosphorylation levels, decrease Cx40 expression, and lessen the severity of TBI (Chen et al., 2016). Furthermore, after brain injury in rats (Male Sprague Dawley rats weighing between 250 and 300 g were subjected to subarachnoid hemorrhage induction using the modified double hemorrhage model, followed by intravenous treatment administration.), GsRb1 can relieve brain edema, weaken basilar artery vasospasm, reduce lumen thickness, and enhance neurobehavior (Li et al., 2011). Besides, autophagy is increased post-TBI, and GsRb1 acts as a neuroprotective agent in TBI rats by suppressing excessive autophagy (Guo et al., 2014), reducing neurological dysfunction scores, and ameliorating neurological damage (Zou et al., 2022). Traditional Chinese Medicine (TCM) has been receiving attention as an effective treatment for TBI, but anti-apoptotic and anti-autophagic activities of GsRb1 are also neuroprotective against TBI and could be further investigated as a potential therapeutic agent.

### 4.3 Ginsenoside Rb1 and spinal cord injury

Spinal cord injury (SCI) causes severe damage to the central nervous system, resulting in sensorimotor dysfunction of the trunk and extremities (Fan et al., 2018). Approximately 70% of SCI patients can develop chronic neuropathic pain (NP), which can manifest as abnormal pressure pain, nociceptive hypersensitivity (increased sensitivity to non-injurious stimuli), nociceptive hypersensitivity (increased sensitivity to injurious stimuli), or spontaneous pain (Finnerup, 2013; Dosenovic et al., 2017). The induction and maintenance of NP are importantly linked to the activation of MAPK (p38MAPK, ERK, and JNK) in glial cells (Hains and Waxman, 2006; Choi et al., 2012; Jendelova, 2018), and GsRb1 can exert an anti-apoptotic effect, inhibit this pathway, and ameliorate the clinical manifestations of NP. Inflammatory cytokines and mediators contributing to SCI-induced NP include IL-6, inducible nitric oxide synthase (iNOS), and COX-2, produced by activated glial cells (Zhang and De Koninck, 2006; Detloff et al., 2008; Jendelova, 2018). Studies have demonstrated that GsRb1 can lessen neuronal damage and enhance neurological function in SCI models by lowering neuronal apoptosis, blocking autophagy, attenuating oxidative stress, and encouraging the restoration of motor function (Huang et al., 2015; Bisicchia et al., 2017; Wang et al., 2018; Wen et al., 2023). Additionally, a study discovered that GsRb1 reduced the amplitude of nerve action potentials to treat cervical spondylosis similar to SCI and heal spinal cord damage (Lee et al., 2021). GsRb1 and its metabolite CK can be used in the clinic as an anti-injury (reduction of mechanical, cold, and thermal nociceptive hypersensitivity) drug to alleviate NP symptoms through the endoplasmic reticulum-mediated expression of estrogen receptor (ER)-α (ER-α) and ER-β expressed in the dorsal horn neurons of the L4-L5 spinal cord (Lee et al., 2021; Shoaib et al., 2023), which provides new insight into the future strategy for the diagnosis and treatment of SCI in humans.

### 4.4 Ginsenoside Rb1 and Parkinson’s disease

Dyskinesia and non-motor symptoms are the hallmarks of PD—a chronic, progressive neurodegenerative condition (Mahoney-Sánchez et al., 2021). From a pathophysiological perspective, PD is primarily characterized by neurodegeneration coupled with misfolding and aggregation of the neuronal inclusions α-synuclein (α-Syn), which accumulate into protein inclusions within the cell and lead to the formation of Lewy bodies, as well as the loss or degeneration of nigrostriatal dopaminergic neurons (Beitz, 2014; Bridi and Hirth, 2018; Dickson, 2018; Degirmenci et al., 2023). A key etiology of PD is an imbalance between the excitatory Glu and inhibitory γ-aminobutyric acid (GABA) systems, which results in neuro excitotoxicity and dopaminergic cell death in the substantia nigra compacta (Rose, 2016). Gamma-aminobutyric acid A receptor alpha 1 (GABAARα1) may interact with GsRb1 through hydrophobic interactions to upregulate the expression of GABAARα1 in cells, which enhances GABAergic transmission and reduces the dysfunction of GABA-mediated inhibitory transmission (Liu et al., 2019). In mice (Ten-week-old male C57BL/6 mice were obtained from SLAC Laboratory Animal Co., Ltd., Shanghai, China.), GsRb1 showed increased postsynaptic density protein 95 (PSD-95) expression in an α-Syn-dependent manner *in vitro* and *in vivo*, slowed memory loss and long-term potentiation (LTP) induced by 1-Methyl-4-phenyl-1,2,3,6-tetrahydropyridine (MPTP) (which induces typical parkinsonism in both humans and primates), whereas GsRb1 alleviated glutamate excitotoxicity by upregulating glutamate transporter expression and treated memory impairment and dyskinesia in PD patients with PD (Zhang YL. et al., 2018; Qu et al., 2019).

Furthermore, Ahmed T et al. discovered that GsRb1 affects the length of neural protrusions and increases the lifespan of neurons, which further supports the idea that GsRb1 has a beneficial therapeutic effect on neurodegenerative illnesses (Ahmed et al., 2016). Memory loss is a non-motor symptom of PD, and the hippocampus of the brain has a memory processing storage function and spatial information processing function, and the protection of hippocampal function is crucial to treating PD (Györfi et al., 2018). Lei Liu et al. and Qu S et al. demonstrated that reduced α-Syn in the hippocampal CA3 region causes memory deficits and that oral administration of GsRb1 significantly increases cell survival in the dentate gyrus and hippocampal CA3 region as one of the mechanisms for improving spatial learning and memory (Liu et al., 2011; Qu et al., 2019). As a novel neurotarget therapy for PD, GsRb1 affects hippocampal neurons in the brain by selectively inhibiting L-type calcium channels (Lin et al., 2012; Liss and Striessnig, 2019). PD is an untreatable condition that develops gradually over time. GsRb1 may be a possible medication to treat PD, owing to its capacity to ameliorate symptoms and enhance patients’ quality of life. Pharmacological treatment is the first option for Parkinson’s patients and is the cornerstone of the total treatment process.

### 4.5 Ginsenoside Rb1 and Alzheimer’s disease

The pathomechanism of AD—a neurodegenerative condition that impairs cognitive function in the brain—is characterized by the development of amyloid plaques, neurofibrillary tangles (NFTs), and significant neuronal loss (Selkoe, 2001; Town, 2010). According to studies, beta-amyloid peptides can cause tau protein (the microtubule-associated protein) to phosphorylate abnormally, which causes microtubule destabilization, poor axonal transport, and ultimately neuronal death (Zheng et al., 2002; Huang and Jiang, 2009). GsRb1 reduces calpain and p25 expression levels and attenuates β-amyloid peptide (25–35)-induced tau hyperphosphorylation in cortical neurons via the cyclin-dependent kinase 5 (CDK5) pathway, which promotes increased microtubule stability, intracellular calcium homeostasis in neuronal cells, neuronal cell protection and has significant potential to treat AD (Humbert et al., 2000; Chen et al., 2008; Shalaby et al., 2023; Sharari et al., 2023). In a study involving mice (Healthy male Sprague Dawley rats (clean grade) with a weight range of 300–320 g were supplied by the Animal Center of the First Hospital affiliated with Harbin Medical University, located in Harbin, China.) AD models and β-amyloid peptide (25–35), GsRb1 significantly improved axonal atrophy, synaptic loss, and memory impairment (Tohda et al., 2004). By contrast, another study demonstrated that GsRb1 could encourage neural stem cell proliferation and differentiation in a rat (Alzheimer’s disease animal models were induced by injecting Amyloid beta 1–40 in healthy male Sprague Dawley rats, 6 weeks old, from SLC, Shizuoka, Japan.) model of AD (Zhao J. et al., 2018), indicating that GsRb1 may have several therapeutic roles in AD, delaying AD progression and enhancing the patient’s quality of life. Oxidative stress plays a significant role in the pathogenesis of neurological disorders; thus, GsRb1 may offer fresh perspectives on preventing and treating AD by encouraging reverse cholesterol transport and reducing ROS formation (Changhong et al., 2021). Neuroinflammation plays a substantial role in AD etiology (Leng and Edison, 2021), which can activate neurodegenerative signaling pathways and promote plaque aggregation (Zhang W. et al., 2023). GsRb1 exerted anti-neuroinflammatory effects and corrected the loss of learning and memory skills in a rat (Male Wistar rats weighing 250–300 g and aged 3–4 months old were used to create Alzheimer’s disease rat models through intracerebroventricular injection of Amyloid beta 1–42.) model of AD (Wang et al., 2011; Jiang et al., 2023). Currently, GsRb1 can protect the nerves, enhance cognitive function, and be applied in preclinical and clinical studies of AD through several administration methods. Drug delivery to the central nervous system remains a complex process (Passeri et al., 2022), and there has been no improved treatment for AD.

### 4.6 Ginsenoside Rb1 and epilepsy

Epilepsy is a chronic disease with transient disorders of brain function. A significant cause of seizures is the imbalance between excitatory and inhibitory neurotransmission (Amtul and Aziz, 2017). The onset of the disease is characterized by sudden abnormal discharges of neurons in the brain. The type of the disease onset is linked to the patient’s genetics and the environment. The oxidative stress in the body and the impairment of autophagy can also lead to epileptic seizures (Berkovic et al., 2006; Mcmahon et al., 2012). In a rat (Healthy male Sprague-Dawley rats, with a weight range of 220–240 g, were sourced from the Animal Experimental Center of Zhengzhou University.) study, researchers used the antiepileptic drug γ-aminobutyric acid receptor antagonist pentylenetetrazole to mimic epilepsy and then treated with GsRb1, demonstrating that MDA and Bcl-2 expressions were upregulated during seizures and that the expressions of GSH, iNOS, and LC3 were downregulated, suggesting that GsRb1 acts as an inhibitor of oxidative stress, autophagy, and apoptosis, and attenuates neurological damage during seizures in epileptic rats (Shi et al., 2018; Zeng et al., 2018). GsRb1 also increases the *in vivo* and *in vitro* expression of Nrf2 and heme oxygenase-1 (HO-1) and protects the nerves by activating the nuclear factor erythroid 2-related factor 2/antioxidant response element (Nrf2/ARE) signaling pathway, reducing oxidative stress and neuronal apoptosis, shortening seizure duration while prolonging the latency period for seizure reoccurrence, thereby reducing brain damage during seizures, decreasing seizure severity, and improving cognitive dysfunction (Shi et al., 2018; Chen et al., 2019). At the moment, drug-based treatment is the only option available to stop the development or progression of epilepsy (Xu et al., 2013). Identifying effective medications to lower the frequency of seizures is necessary because treating recurrent seizures is currently difficult. GsRb1 can be used as a new antiepileptic medication as it contains anti-inflammatory, anti-apoptotic, and neurogenesis-inducing properties that can prevent neuronal damage, lessen the frequency and severity of seizures, and improve cognitive impairment.

## 5 Conclusion

This comprehensive review focuses on GsRb1, a natural compound with significant medicinal potential. A thorough analysis of its pharmacokinetic data provides a comprehensive understanding of the metabolic pathways and kinetic characteristics of GsRb1 in the human body, which is crucial for optimizing its clinical applications and drug development. Additionally, we delve into the actions and mechanisms of GsRb1 in the human body, such as its roles in suppressing inflammation, oxidative stress, apoptosis, and autophagy. By elaborating on how GsRb1 modulates multiple biomolecules and signaling pathways, we offer an in-depth insight into the compound’s multifaceted mechanisms at the cellular level. This comprehensive understanding can inspire new research directions and perspectives, including the development of multi-target drugs. Given GsRb1’s involvement in multiple signaling pathways, exploring its potential as a multi-target drug is not only feasible but also particularly promising for diseases that require the simultaneous regulation of various physiological processes, such as neurodegenerative diseases or autoimmune disorders. Furthermore, we can translate these cellular-level findings into clinical research and human trials in the future to evaluate the effectiveness and safety of GsRb1 in practical treatments. These considerations can significantly expand our understanding of GsRb1, providing fresh perspectives and insights for future research and clinical applications.

GsRb1 plays an indispensable role in the nervous system. Its potential for neuroprotection and treatment has garnered widespread attention in the scientific community, offering new hope for research and clinical treatment of neurological diseases. This comprehensive review aims to provide researchers and clinicians with a rich resource for understanding GsRb1, promoting further research and application. It holds profound significance for current scientific research and medical practice. The aforementioned studies have some limitations. There have been few preclinical efficacy assessments of GsRb1, and further research is required to determine whether the drug’s mechanism of action in cellular or animal models is entirely transferable to people. Second, additional research is warranted to determine whether GsRb1 causes other systemic side effects when used to treat neurological disorders because GsRb1 has some targets binding to the cardiovascular and endocrine systems.

## Figures and Tables

**Table:** 

**α-Syn**	α-synuclein
**AD**	Alzheimer’s disease
**Akt/PKB**	Protein kinase B
**Arg-I**	Arginase-I
**ARE**	Antioxidant response element
**Asp**	Aspartate
**AUC**	Area under the curve
**BBB**	Blood-Brain Barrier
**Bcl-2**	B-cell lymphoma 2
**Bcl-XL**	B-cell lymphoma-extra large
**BDNF**	Brain-derived neurotrophic factor
**BECN1**	Beclin1
**BLB**	Blood-Labyrinth Barrier
**CA**	Cornu ammonis
**Ca** ^**2+**^	Calcium ions
**cAMP**	Cyclic adenosine monophosphate
**CDK5**	Cyclin-dependent kinase 5
**CK**	Compound K
**Cmax**	Maximum concentration
**COX-2**	Cyclooxygenase-2
**CREB**	cAMP response element-binding protein
**Cx40**	Connexin 40
**dgRb1**	Dihydroginsenoside Rb1
**DNA**	Deoxyribonucleic acid
**ER**	Estrogen receptor
**ERK**	Extracellular regulated protein kinase
**GABA**	γ-aminobutyric acid
**GABAARα1**	Gamma-Aminobutyric acid type A receptor Alpha 1 subunit
**GDNF**	Glial cell-derived neurotrophic factor
**GLT-1**	Glutamate transporter 1
**Glu**	Glutamate
**GSH**	Scavenger glutathione
**GsF2**	Ginsenoside F2
**GsRb1**	Ginsenoside Rb1
**GsRd**	Ginsenosides Rd
**HMGB1**	High mobility group box protein 1
**HO-1**	Heme oxygenase-1
**I-κB**	Nuclear factor kappa-B kinase subunit Alpha
**iNOS**	Inducible nitric oxide synthase
**IL**	Interleukin
**IV**	Intravenous
**IT**	Intratympanic
**JNK**	C-Jun N-terminal kinase
**LC3**	Light chain 3
**LTP**	Long-term potentiation
**MAPK**	Mitogen-activated protein kinase
**MDA**	Malondialdehyde
**MPP+**	1-methyl-4-phenylpyridine
**MPTP**	1-Methyl-4-phenyl-1,2,3,6-tetrahydropyridine
**MRT**	Mean residence time
**mTOR**	Mammalian target of rapamycin
**NADPH**	Nicotinamide adenine dinucleotide phosphate
**NF-κB**	Nuclear factor-kappa B
**NFTs**	Neurofibrillary tangles
**NO**	Nitric oxide
**NOX**	Nicotinamide adenine dinucleotide phosphate oxidase
**NP**	Neuropathic pain
**Nrf2**	Nuclear factor erythroid 2-related factor 2
**Nurr1**	Nuclear receptor-associated protein 1
**OS**	Oxidative stress
**p38 MAPK**	p38 mitogen-activated protein kinases
**p62**	Sequestosome 1
**p-Akt**	Phosphorylated-Akt
**PC12**	Pheochromocytoma cells
**PD**	Parkinson’s disease
**P-gp**	P-glycoprotein
**pH**	Potential of hydrogen
**PI3K**	Phosphoinositide 3-kinase
**PKA**	Protein kinase A
**p-mTOR**	Phosphorylated-mTOR
**PNS**	Panax notoginseng saponins
**p-PTEN**	Phosphorylated-PTEN
**PSD-95**	Postsynaptic density protein 95
**PTEN**	Phosphatase and Tensin Homolog
**ROS**	Reactive oxygen species
**SAPK**	Stress-activated protein kinase
**SCI**	Spinal cord injury
**SIRT1**	Silent information regulator 1
**SOD**	Superoxide dismutase
**t**_**1/2**_	Half-life
**TBI**	Traumatic brain injury
**TCM**	Traditional Chinese medicine
**TNF-α**	Tumor necrosis factor-α
